# Somatic Mosaicism of IDH1 R132H Predisposes to Anaplastic Astrocytoma: A Case of Two Siblings

**DOI:** 10.3389/fonc.2019.01507

**Published:** 2020-01-14

**Authors:** Sulgi Lee, Madhuri Kambhampati, M. Isabel Almira-Suarez, Cheng-Ying Ho, Eshini Panditharatna, Seth I. Berger, Joyce Turner, David Van Mater, Lindsay Kilburn, Roger J. Packer, John S. Myseros, Eric Vilain, Javad Nazarian, Miriam Bornhorst

**Affiliations:** ^1^Center for Genetic Medicine, Children's National Health System, Washington, DC, United States; ^2^Institute for Biomedical Sciences, The George Washington University School of Medicine and Health Sciences, Washington, DC, United States; ^3^Department of Pathology and Laboratory Medicine, Children's National Health System, Washington, DC, United States; ^4^Department of Pathology and Neurology, University of Maryland School of Medicine, Baltimore, MD, United States; ^5^Pediatric Oncology, Dana-Farber Cancer Institute, Boston, MA, United States; ^6^Department of Genomics and Precision Medicine, The George Washington University School of Medicine and Health Sciences, Washington, DC, United States; ^7^Rare Disease Institute, Children's National Health System, Washington, DC, United States; ^8^Department of Pediatrics, The George Washington University School of Medicine and Health Sciences, Washington, DC, United States; ^9^Division of Genetics and Metabolism, Children's National Health System, Washington, DC, United States; ^10^Division of Oncology, Children's National Health System, Washington, DC, United States; ^11^Division of Pediatric Hematology Oncology, Department of Pediatrics, Duke University Medical Center, Durham, NC, United States; ^12^Brain Tumor Institute, Children's National Health System, Washington, DC, United States; ^13^Division of Neurosurgery, Children's National Health System, Washington, DC, United States; ^14^University Children's Hospital Zurich, Zurich, Switzerland

**Keywords:** anaplastic astrocytoma, mosaicism, cancer predisposition, ddPCR, AYA (adolescents and young adults), IDH1 R132H mutation

## Abstract

Anaplastic astrocytomas are aggressive glial cancers that present poor prognosis and high recurrence. Heterozygous IDH1 R132H mutations are common in adolescent and young adult anaplastic astrocytomas. In a majority of cases, the IDH1 R132H mutation is unique to the tumor, although rare cases of anaplastic astrocytoma have been described in patients with mosaic IDH1 mutations (Ollier disease or Maffucci syndrome). Here, we present two siblings with IDH1 R132H mutant high grade astrocytomas diagnosed at 14 and 26 years of age. Analysis of IDH^R132H^ mutations in the siblings' tumors and non-neoplastic tissues, including healthy regions of the brain, cheek cells, and primary teeth indicate mosaicism of IDH^R132H^. Whole exome sequencing of the tumor tissue did not reveal any other common mutations between the two siblings. This study demonstrates the first example of IDH1 R132H mosaicism, acquired during early development, that provides an alternative mechanism of cancer predisposition.

## Background

Gliomas comprise nearly one-third of newly diagnosed brain tumors in adolescents and young adults (AYA; 15–39 years) (1). While most AYA gliomas are low grade (grade I/II), patients who present with anaplastic astrocytomas and glioblastomas (grade III/IV) have high risk of recurrence and poor prognosis (2). Heterozygous mutations in *IDH1* genes are common alterations in AYA gliomas, particularly in grade II diffuse astrocytomas, anaplastic astrocytomas, secondary glioblastomas, and oligodendrogliomas (3, 4). The most frequent of these mutations is the *IDH1 c.395 G*>*A* (IDH1 R132H) mutation. Genetic studies suggest that *IDH1* mutations are an early clonal event in anaplastic astrocytoma development (5, 6). Mutations in *IDH1* gene result in the production of 2-hydroxyglutarate (2HG) from alpha-ketoglutarate. The high levels of 2HG in cells promote histone methylation and block differentiation of progenitor cells, creating an environment that encourages the accumulation of additional mutations, such as *TP53* and *ATRX*, and thus resulting in unchecked cellular proliferation (7).

Familial gliomas are rare, and have primarily been described in patients with inherited or sporadic germline cancer predisposition syndromes such as Neurofibromatosis Type 1 (*NF1*), Li-Fraumeni syndrome (*TP53*), hereditary breast/ovarian cancer syndrome (*BRCA2*), constitutional mismatch repair deficiency syndrome (*MLH1, MSH2, MSH6*, and *PMS2)*, Turcot syndrome (*APC*), Tuberous sclerosis (*TSC1/TSC2*), Melanoma-neural system tumor syndrome (*p16/CDKN2A*), and *POT1*-associated familial gliomas (8–10). Inherited risk variants, such as those near the genes *TERC, TERT, EGFR, CDKN2B, PHLDB1, RTEL, TP53*, and *CCDC26*, may also contribute to glioma formation (10–15). Of these, patients with inherited risk variants near *CCDC26* (allele G in rs55705857) have the highest risk of developing *IDH1/2*-mutant gliomas, although tumor penetrance in patients who carry these variants is low (14). Somatic mosaic mutations can also predispose to glioma development. Patients with Ollier disease and Maffucci syndrome, which are rare enchondromatosis syndromes, have been shown to carry mosaic mutations of *IDH1*, including IDH^R132H^ (16, 17). The most common tumors in patients with *IDH1* mosaic mutations are multiple enchondroma and cavernous hemangiomas (Maffucci), but anaplastic astrocytomas have also been reported (17, 18). Recent studies have shown that somatic mosaicism resulting from mutations acquired during embryonic development can influence the likelihood of developing cancer in adulthood (19).

## Materials and Methods

### Ethics Approval and Consent for Publication

This study was approved and carried out in accordance with Children's National Hospital's Institutional Review Board (IRB #1339 and #6778). All adult subjects, and in the case of sibling A (minor) his parents, gave written informed consent in accordance with the Declaration of Helsinki to the publication of results and the collection and analysis of specimens utilized in this study including post-mortem whole brain of sibling A, resected tumor specimens, whole blood and cheek swab of sibling B, whole blood and cheek swabs of family members, healthy-donor teeth.

Please see Supplementary Materials for additional materials and methods used in this publication.

## Case Presentation

### Clinical Presentation of Siblings With IDH1 R132H Mutant Astrocytoma

**Figure 1:**
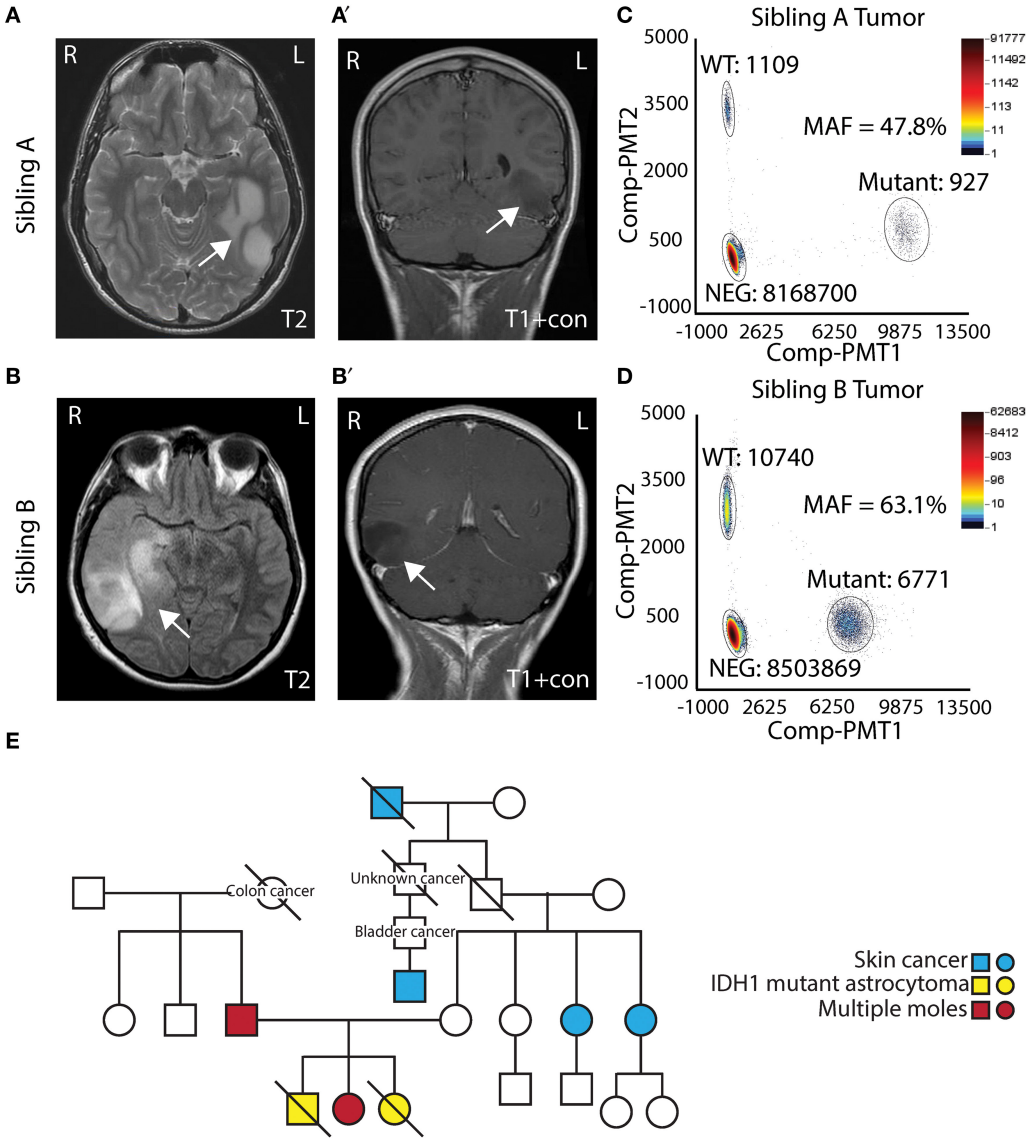
Clinical presentation of siblings with IDH1 R132H mutant astrocytoma. **(A,A′)** T2 and T1 contrast MRI showing a left temporal mass in Sibling A. **(B,B′)** T2 and T1 contrast MRI showing a right temporal mass in Sibling B. **(C)** Digital droplet PCR (ddPCR) analysis of Sibling A's tumor showing mutation allelic frequencies of 47.8%. **(D)** Digital droplet PCR (ddPCR) analysis of Sibling B's tumor showing allelic frequencies of 63.1%. **(E)** Pedigree of the family.

Two AYA siblings presented with anaplastic astrocytoma. Sibling A (Male) presented at 14 years of age with a large non-enhancing left temporal mass (Figures 1A,A′) and a pathological diagnosis of Grade III/IV astrocytoma. Treatment for his tumor included radiation therapy, chemotherapy (Temozolamide + metronomic chemotherapy), and vaccine therapy through a clinical trial. Despite therapy, his tumor continued to progress, and he passed away 5 years after diagnosis. His family opted to donate his whole brain and tumor to the Children's Research Institute at Children's National Health System at post mortem. Sibling B (Female) presented to medical attention after experiencing a seizure, 1 year after sibling A passed away. She was also noted to have a right temporal lobe non-enhancing mass (Figures 1B,B′). She was initially treated with surgery alone, and pathology showed a Grade II/III astrocytoma. At recurrence, 1 year after diagnosis, pathology revealed a more aggressive Grade III/IV astrocytoma. She had surgery followed by radiation and chemotherapy (Temozolamide + metronomic therapy). She developed additional tumors in the pons and spinal cord, and she passed away just over 2 years following her diagnosis. Biospecimens were not available for post-mortem analysis.

Both sibling A and sibling B's tumors were noted to have IDH^R132H^ mutant protein expression on IHC. To assess the allelic frequency of the mutation, DNA was obtained from frozen tumor (both cases) and analyzed by ddPCR. Analysis showed mutation allelic frequencies of 47.8% (for sibling A) and 63.1% (sibling B) indicating the heterozygous status of *IDH1 c.395 G*>*A* (IDH^R132H^) mutation (Figures 1C,D). Given similarities in tumor pathology and their presentation, a cancer predisposition syndrome was strongly suspected. Sibling B was evaluated in cancer genetics clinic at Children's National where three generation pedigree was obtained and did not suggest a specific familial cancer predisposition syndrome on the maternal or paternal side of the family (Figure 1E). Clinical whole exome trio sequencing of blood DNA from sibling B and her parents was performed. A maternally inherited *PTCH2* variant of unclear significance was identified, but no pathogenic mutations pertaining to inherited glioma predisposition were detected.

### Temporal Detection of IDH1 R132H in Sibling a at Post Mortem

**Figure 2:**
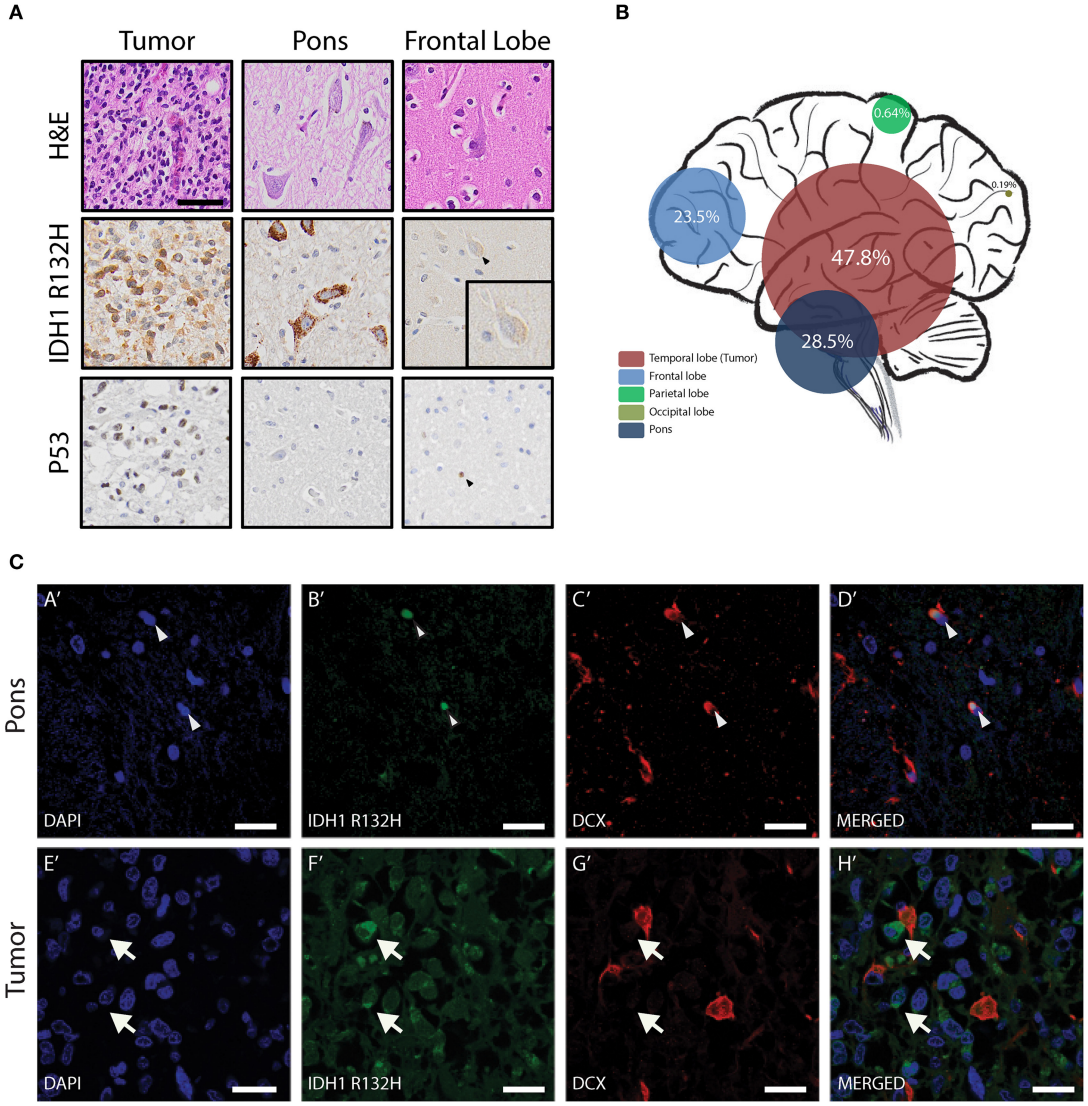
Temporal detection of IDH1 R132H in Sibling A at post mortem. **(A)** Immunohistochemical analysis of post-mortem brain of Sibling A indicating presences of IDH^R132H^ mutant cells in the tumor and other non-neoplastic regions of the brain. p53 immunohistochemistry confirms that there are no infiltrating tumor cells in other regions of the brain (scale bar = 50 μm). **(B)** Mutation allelic frequency of IDH^R132H^ detected in frontal lobe, temporal lobe, parietal lobe, occipital lobe, and pons by ddPCR. **(C)** Immunofluorescent staining of the pons indicating co-localization of IDH^R132H^ and DCX (arrowheads in **D′**). Similar pattern of co-localization of IDH^R132H^ and DCX is absent in the tumor (arrows in **H′**) (scale bar = 30 μm).

To further investigate potential genetic similarities between the two siblings, DNA was extracted from both tumors to be assessed by whole exome sequencing (WES). Sibling A did not have whole blood, cheek swab, or skin DNA to use as a normal control; hence the only available source of germline DNA was the unaffected postmortem brain tissue. Recognizing the diffuse nature of these tumors, both IHC and ddPCR for the IDH^R132H^ mutation were performed on non-neoplastic brain regions (as indicated by MRI), including right pons, frontal, parietal, and occipital lobes. IHC and ddPCR indicated the presence of IDH^R132H^ mutation in all the tested regions (Figures 2A,B). IDH1^R132H^ was seen in only a fraction of the cells in these regions indicating the variation is mosaic and not in every cell. Interestingly, in the tumor most of the IDH1^R132H^ positive cells had morphology consistent with glia cells (Figure 2A), but in the other regions of the brain, such as the pons and the frontal lobe, the IDH1^R132H^ positive cells primarily showed features that resembled neurons (Figure 2A top and middle panels). Although not all IDH mutant astrocytic tumors co-occur with mutations or alterations in p53, since the patient's tumor cells showed p53 protein expression by IHC (Figure 2A bottom panel), IHC for p53 was performed on all the other regions of the brain, to determine if the IDH^R132H^ mutant cells are infiltrating tumor cells. All the regions tested were negative for p53 except the frontal lobe, where rare p53-positive glia cells were identified (Figure 2A lower panel, arrowhead). Immunofluorescent staining revealed co-localization of IDH^R132H^ mutation and DCX, an immature neuron marker, in the pons (Figures 2CA′–D′) confirming that IDH^R132H^ positive cells are indeed young neurons and not infiltrating tumor cells. This pattern of IDH^R132H^ and DCX co-localization was not seen in sibling A's tumor (Figures 2CE′–H′).

### IDH1 Mosaicism Revealed by Presence of IDH1 R132H in Other Biospecimens

**Figure 3:**
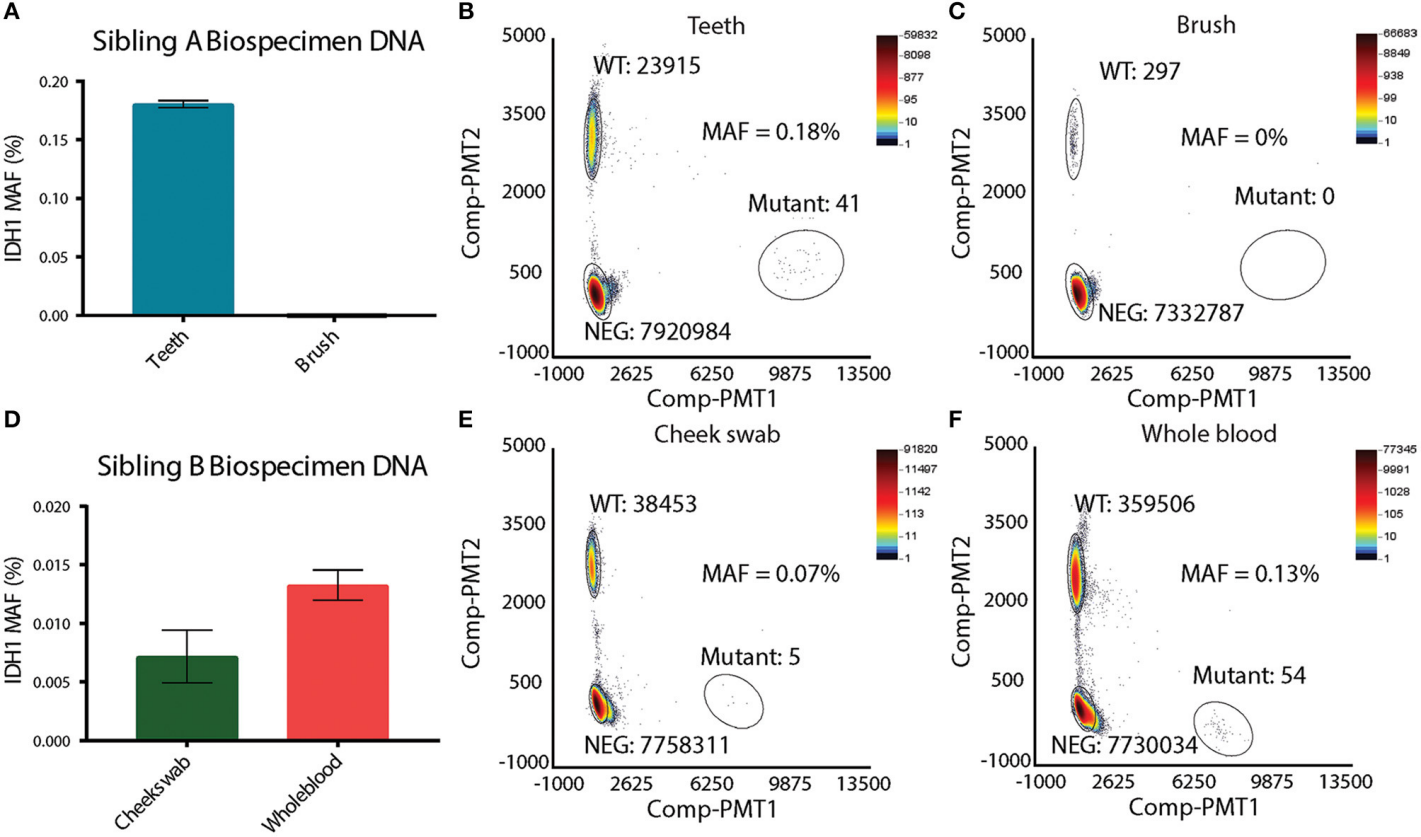
Presence of IDH1 R132H in other biospecimens. **(A)** Mutation allelic frequency of IDH^R132H^ detected in Sibling A's teeth and toothbrush by ddPCR. **(B)** ddPCR plot of Sibling A's tooth, showing the copy number of wild-type IDH1 and copy number of IDH^R132H^. **(C)** ddPCR plot of Sibling B's toothbrush, showing the copy number of wild-type IDH1 and the absence of IDH^R132H^. **(D)** Mutation allelic frequency of IDH^R132H^ detected in Sibling B's cheek swab and whole blood by ddPCR. **(E)** ddPCR plot of Sibling B's cheek swab, showing the copy number of wild-type IDH1 and copy number of IDH^R132H^. **(F)** ddPCR plot of Sibling B's whole blood, showing the copy number of wild-type IDH1 and copy number of IDH^R132H^.

Suspecting that sibling A has a unique mosaic pattern of IDH^R132H^ mutant cells, alternative specimens that would provide germline DNA were requested from the family. Sibling A's toothbrush (last used 4 years prior to analysis) and primary deciduous teeth were obtained. The toothbrush was processed in a Tris base solution prior to DNA extraction (see Methods and Figure S1A). DNA from deciduous teeth was extracted by pulverization with liquid nitrogen (see Methods and Figure S1B). DNA from deciduous teeth was quantified and showed a linear relationship between the tooth weight and the amount of DNA isolated (Figures S1C,C′). In order to assess the feasibility of detecting single nucleotide variation in DNA isolated from the teeth and toothbrush, ddPCR using probes for wild-type histone *H3F3A* was performed. The *H3F3A* histone DNA was detected in both the teeth and toothbrush DNA (Figures S1D,E). As expected, there were significantly fewer DNA copies in the toothbrush (Figure S1E) than in the teeth samples (Figure S1D). DNA isolated from the teeth and the toothbrush of sibling A was then analyzed for the IDH1^R132H^ mutation via ddPCR. The toothbrush DNA did not contain any IDH1^R132H^ mutant copies, which was likely affected by the very low levels of DNA (Figures 3A,C). The teeth DNA showed IDH1^R132H^ copies at average mutant allelic frequency (MAF) of 0.18% (Figures 3A,B). To eliminate possible contamination or experimental errors, ddPCR on DNA extracted from teeth donated by 7 healthy individuals was analyzed to validate the findings (Figure S2A). As expected, IDH1^R132H^ mutant copies were not detected, validating the sensitive detection of an IDH1^R132H^ mutation by ddPCR in the deciduous teeth DNA of sibling A. This finding, along with the IDH1^R132H^ mutation pattern in the brain, supports the presence of IDH1^R132H^ somatic mosaicism in sibling A.

### Detection of IDH1 R132H in Tumor and Indications of Somatic Mosaicism in Sibling B

**Figure 4:**
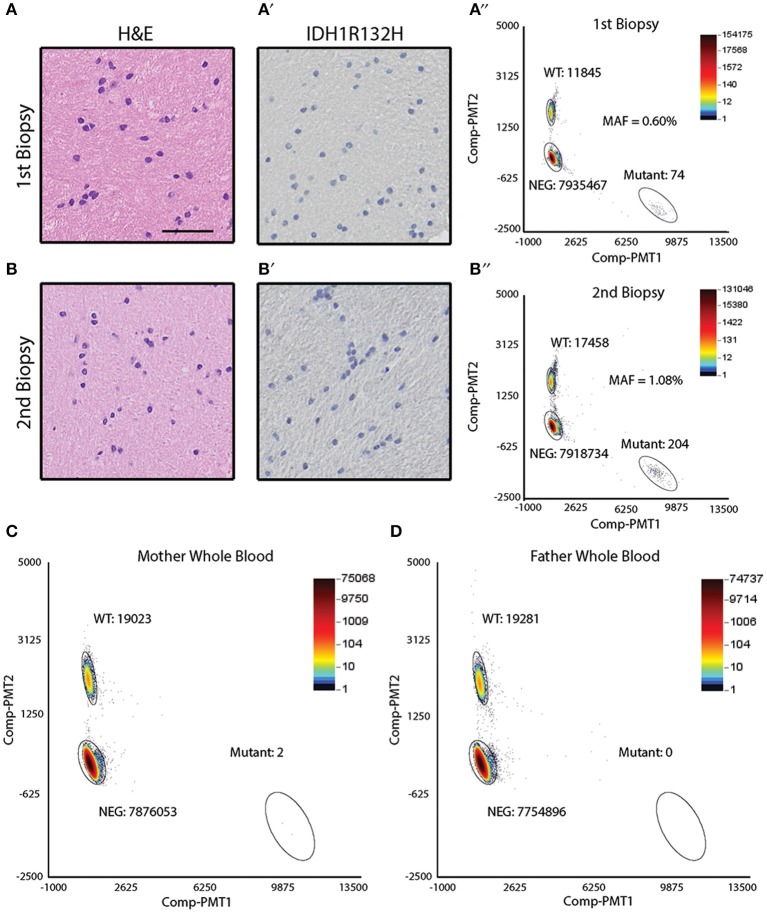
Detection of IDH1 R132H in the frontal lobe biopsy of sibling B and parental whole blood. **(A)** Hematoxylin & eosin staining of the biopsied frontal lobe tissue shows reactive gliosis (scale bar = 50 μm). **(A′)** Immunohistochemical analysis of the biopsied frontal lobe tissue shows absence of IDH^R132H^ mutant cells. **(A″)** ddPCR analysis of the DNA isolated from the biopsied frontal lobe tissue shows IDH^R132H^ mutational allelic frequency of 0.60%. **(B)** Hematoxylin & eosin staining of the biopsied frontal lobe tissue shows hyperplasia. **(B′)** Immunohistochemical analysis of the biopsied frontal lobe tissue shows absence of IDH^R132H^ mutant cells. **(B″)** ddPCR analysis of the DNA isolated from the biopsied frontal lobe tissue shows IDH^R132H^ mutational allelic frequency of 1.08%. **(C)** ddPCR analysis of maternal whole blood shows mutant allelic frequency average of 0.007%. **(D)** ddPCR analysis of paternal whole blood shows absence of IDH^R132H^ mutant copies.

Sibling B was diagnosed with an astrocytoma of the right temporal lobe (Figure 1B). During the course of her treatment, sibling B had two biopsies of a suspicious region (T2 bright on MRI) in the left frontal cortex that showed reactive gliosis (biopsy 1) and hyperplasia (biopsy 2) (Figures 4A,B), but no obvious tumor. Both biopsy specimens were noted to be IDH^R132H^ negative by IHC (Figures 4A′,B′). DNA was extracted from the biopsy specimens to perform ddPCR for IDH^R132H^. IDH^R132H^ was detected with an MAF of (0.60%) and (1.08%) for biopsy 1 and 2, respectively (Figures 4A″,B″). This supports that sibling B also had a low level of IDH^R132H^ mosaicism in non-neoplastic brain regions.

To test whether sibling B exhibited a similar pattern of somatic mosaicism as sibling A, DNA isolated from cheek swabs and whole blood DNA was examined. Interestingly, the cheek swab and whole blood of sibling B revealed IDH^R132H^ mutant copies at an average MAF of 0.07 and 0.13%, respectively, indicating a similar mosaic pattern to that observed in the other germline biospecimens of sibling A (Figures 3D–F). To further confirm this finding, DNA from deciduous teeth of sibling A, and DNA from a cheek swab of sibling B were analyzed by next generation sequencing (NGS). This data confirmed the ddPCR findings, where IDH^R132H^ mutant copies were present at very low levels (MAF of 0.29%, and 0.01% for teeth of sibling A and cheek swab of sibling B, respectively) (Figures S2B,C).

Since IDH^R132H^ mosaicism in two siblings is rare, other potential explanations for the findings were considered. Our laboratory has previously shown that H3.3K27M somatic mutations can be found in circulating tumor DNA (plasma) of children diagnosed with DIPG (20). Similarly, IDH^R132H^ mutations can be detected in CSF, and less consistently in plasma of patients with IDH^R132H^ positive tumors using ddPCR (21, 22). In order to rule out the presence of circulating tumor DNA in the cheek swab specimen obtained from sibling B, cheek swabs from two patients diagnosed with diffuse intrinsic pontine glioma (DIPG) with known H3.3K27M mutations (as confirmed by tumor biopsy) were collected. ddPCR was performed on the DNA extracted from the cheek swabs and matched plasma specimens. Despite positive results in the plasma, the H3.3K27M mutation was not found in the cheek swabs from these patients (Figure S3A). Since patients with IDH^R132H^ mutant tumors may behave differently than patients with DIPG, ddPCR was also performed on cheek swab DNA from two additional patients diagnosed with IDH^R132H^ positive tumors (one with a progressive tumor, and the other with multifocal tumors). Cheek swab samples from these two patients were negative for IDH^R132H^ by ddPCR analysis (Figure S3B). In summary, the findings suggest that this is a case of IDH^R132H^ somatic mosaicism in two siblings, predisposing them to the development of *IDH1*-mutant glial tumors.

### Detection of IDH1 R132H in Maternal Blood

Given the possibility of genetic or environmental exposures to developing the IDH^R132H^ mutation, ddPCR was performed on parental (mother and father) blood samples. The variant was not detected in paternal blood but was identified at low levels (0.007%) in the maternal blood sample (Figures 4C,D). Cheek swabs from both parents did not contain the IDH^R132H^ variant (data not shown).

## Discussion

Familial gliomas without obvious genetic predisposition are rare, and when they are reported, the underlying etiology often remains a mystery (23). In this case, two siblings, one male, and one female, both had IDH^R132H^ mutant anaplastic astrocytomas without obvious genetic predisposition. Although common inherited risk variants could not be assessed, since whole genome sequencing results on either sibling was not available, shared exonic germline mutations associated with familial gliomas were not identified through clinical trio sequencing of patient B and her parents. Whole exome sequencing of tumor/normal DNA (using frontal lobe DNA from sibling A and blood DNA from sibling B) was performed, and, aside from the IDH^R132H^ mutation, WES did not reveal any other shared mutations in the tumor (Figures S4A–C).

Somatic mosaicism occurs when a select number of cells within a developing embryo acquire a mutation that is not corrected through DNA repair mechanisms and is therefore allowed to persist, creating two distinct populations of cells (24). The phenotypic effect of mosaic mutations is dependent on when the mutation occurs during development, with early mutations affecting a larger number of cells than late mutations. In siblings A and B, the IDH^R132H^ mutation was noted in cells obtained from more than one tissue including blood, cheek cells, healthy brain tissue, and teeth. This evidence, along with the early onset of tumor formation in both patients, strongly suggests that the IDH^R132H^ mutation occurred during embryonic development. Yet, only the brain glia cells, which acquired additional somatic mutations (such as *TP53* and *ATRX*) formed a tumor (Figure 2A, Figures S4B,C).

Somatic mosaicism of the same gene in two siblings is extremely rare, and we were not able to find any reports of this occurring in the literature. Although the exact underlying etiology for the somatic mosaicism in these patients is still unknown, it is possible that environmental exposures may have played a role. It is also possible that there is an underlying genetic predisposition that has not yet been discovered. The somatic mosaicism of this variant in the mother, with low levels identified in her blood but not in her cheek swabs, supports either a maternal environmental exposure or a maternally inherited genetic risk factors predisposing to selection of the IDH^R132H^ mutation. An alternative hypothesis includes persistent fetal microchimerism, where the levels detected in the mother represent persistence of cells from her previous pregnancies.

This case highlights an important concept in cancer predisposition and cancer development by providing direct evidence that low level IDH^R132H^ mosaicism, even in patients without classic Ollier or Maffucci syndrome, can predispose to glioma formation. A similar phenomenon has already been described in patients with somatic mosaic mutations in *TSC1/2, TP53, APC, RB1*, and *BRCA1* who have increased risk of developing cancer in multiple tissue types (19, 25–29). Although the incidence of somatic mosaicism in most of these cancer predisposition genes is not known, a recent report of patients with clinical Tuberous Sclerosis Complex but negative germline *TSC1/2* testing revealed mosaicism in 26 of 53 (49%) patients tested, with 5 patients having an allele fraction of <1% by NGS (29). Since 10–15% of patients with TSC are germline negative, this suggests that up to 7.5% of children with TSC have somatic mosaicism. Without highly sensitive methods such as ddPCR used to detect the low-level mosaicism, the mosaic IDH^R132H^ mutant cells likely would have been missed in sibling A and B. Thus, it is very likely that somatic mosaicism of IDH^R132H^ and other mutations is more common than previously recognized. Although ddPCR from two other patients with IDH^R132H^ mutant tumors did not reveal the mutation (Figure S3B), a larger analysis of multiple patients with IDH^R132H^ mutant tumors would be required to determine if this was truly unique to this sibling pair, or if other patients harbor similar mosaicism.

Increased awareness of early somatic mosaic mutations contributing to cancer development in children and young adults will improve our understanding of tumorigenesis. Therefore, further research is required to tease apart the mechanisms of IDH^R132H^ somatic mosaicism and its contribution to glioma predisposition. Ultimately, recognizing this alternative mechanism of cancer predisposition is critical for early detection and management of patients at risk.

## Data Availability Statement

All datasets generated for this study are included in the article/Supplementary Material.

## Ethics Statement

This study was approved and carried out in accordance with Children's National Hospital's Institutional Review Board (IRB #1339 and #6778). All adult subjects, and in the case of sibling A (minor) his parents, gave written informed consent in accordance with the Declaration of Helsinki to the publication of results and the collection and analysis of specimens utilized in this study including post-mortem whole brain of sibling A, resected tumor specimens, whole blood and cheek swab of sibling B, whole blood and cheek swabs of family members, healthy-donor teeth.

## Author Contributions

SL conducted the experiments, analyzed the data, and wrote the manuscript. MK assisted with the experiments and data analysis, and maintains specimens in the Children's Research Institute, MA-S provided pathology interpretation of the specimens. C-YH provided pathology interpretation and participated in generation of the tumor whole exome sequencing data. EP assisted with the ddPCR experiment and interpretation of results. SB provided bioinformatics analysis of the whole exome sequencing. JT provided genetic counseling services. DV contributed MRI images. LK and RP provided patient care. JM provided patient care and assisted with collection of the tissue samples. EV reviewed the manuscript and provided scientific consultation. JN and MB conceived of the study, supervised the research, and wrote the manuscript. All authors read and approved the final manuscript.

### Conflict of Interest

The authors declare that the research was conducted in the absence of any commercial or financial relationships that could be construed as a potential conflict of interest.

## Abbreviations

AYA: adolescents and young adults
2HG: 2-hydroxyglutarate
DNA: deoxyribonucleic acid
ddPCR: digital droplet polymerase chain reaction
WES: whole exome sequencing
MRI: magnetic resonance imaging
DCX: doublecortin
IHC: immunohistochemistry
MAF: mutation allelic frequency
DIPG: diffuse intrinsic pontine glioma.
