# Supplementary material for: Somatic Mosaicism of IDH1 R132H Predisposes to Anaplastic Astrocytoma: A Case of Two Siblings
Source: Front Oncol. 2020 Jan 14;9:1507. doi: 10.3389/fonc.2019.01507 (PMC6971203; doi:10.3389/fonc.2019.01507)
Supplement: Supplementary file 1 [file Data_Sheet_1.PDF]

Supplementary Material

Methods

Biological Specimens
Post-mortem whole brain of sibling A, resected tumor specimens, whole blood and cheek swab of sibling B, whole blood and cheek swabs of family members and healthy-donor teeth were collected after obtaining informed consent from the patients in accordance with Children's National Health System's Institutional Review Board approval (IRB #1339 and #6778).

Immunohistochemistry
Immunohistochemistry (IHC) was performed on formalin-fixed paraffin-embedded (FFPE) slides (5μm). FFPE slides were deparaffinized and antigen retrieval completed by heat induced epitope retrieval in citrate buffer (pH = 6.0) for 20 minutes. The tissues were then permeabilized in 0.3% Triton X-100 for 15minutes. Blocking was performed for 1 hour in 5% goat serum followed by incubation with anti- IDH^{R132H} primary antibody (1:200, Dianova, Hamburg, Germany) overnight at 4°C. On the next day, the tissues were washed in phosphate buffered saline (PBS) and incubated with biotin-labeled secondary antibody (1:200, Vector Laboratories, Burlingame, CA). After quenching of endogenous peroxidase with quenching solution (0.3% hydrogen peroxide in methanol) for 30 minutes, the tissues were incubated in Avidin Biotin Complex (ABC) solution (Vector Laboratories, Burlingame, CA) for 30 minutes. Immunostaining was performed with 3,3'-diaminobenzidine (DAB) solution (Cell Signaling Technology, Danvers, MA) and countered stained with hematoxylin.

Immunofluorescent staining
Immunofluorescent staining was performed on FFPE slides. FFPE slides were deparaffinized, and antigen retrieval and blocking were completed as above. The tissues were incubated with anti- IDH^{R132H} primary antibody (1:200, Dianova, Hamburg, Germany) and anti-Doublecortin primary antibody (1:500, Abcam, Cambridge, MA) overnight at 4°C , then incubated with fluorescent tagged secondary antibodies (1:500, Invitrogen, Carlsbad, CA) for 1 hour at room temperature. After washing in PBS, the tissues were briefly stained with 4',6-Diamidino-2-phenylindole (DAPI) and imaged with laser scanning confocal microscope (Zeiss, Jena Germany).

Fragmentation of teeth
A porcelain mortar and pestle was pre-chilled on dry ice. The sample was placed in the chilled mortar and liquid nitrogen was poured over the tooth, enough to submerge the sample. Liquid nitrogen was allowed to evaporate before grinding the sample with the pestle. The sample was slowly ground with the pestle in circular motion until fine powder formed. Alternatively, fragmented teeth were placed in pre-filled bead tubes for hard tissue grinding (Omni International, Kennesaw, GA) and homogenized with FastPrep-24 bead beating system (MP Biomedicals, Santa Ana, CA)

De-calcification of teeth
The sample was transferred to a 15 ml conical tube with 10 ml of 1.0 mM ethylenediaminetetraacetic acid (EDTA), pH=8.0. The sample was de-calcified for 24 hours on a tube rotator at 4 °C. After 24 hours of agitation, the sample was centrifuged at 2000 x *g* for 15 minutes. The old EDTA was removed and the sample was re-suspended with fresh EDTA. De-calcification was repeated for 5 days. After 5 days of de-calcification in EDTA, the sample was washed with 50ml of sterile deionized water to remove ions. The sample was centrifuged at 2000 x *g* for 15 minutes and supernatant was removed. Washing procedure was repeated three times.

Toothbrush Preparation for DNA isolation

The toothbrush head was cut using a heated scalpel and placed in a 15ml conical tube with Tris base solution from ORAcollect DNA collection kit (DNA Genotek Inc, Ottawa, ON). The conical tube containing the sample was gently rocked for 30 minutes to wash the bristles. The Tris base solution was removed from the conical tube and used to extract DNA using the QIAamp DNA Mini-Kit (Qiagen, Mississauga, ON).

DNA isolation

Genomic DNA (gDNA) from the samples were extracted with the QIAamp DNA Mini-Kit (QIAGEN, Mississauga, ON), according to manufacturer's recommendations. DNA concentrations were quantified using the *Qubit* 2.0 Fluorometer (Life Technologies, Carlsbad, CA).

Pre-amplification of genomic DNA

Pre-amplification of DNA was performed in 35-µL reactions using 2 ng of gDNA, 1 x Q5 Hot Start High-Fidelity Master Mix (New England Biolabs, Ipswich, MA) and 50 nM of forward and reverse primers for IDH1[5'-GCTTGTGAGTGGATGG-3' (forward) and 5'-CATGCAAAATCACATTATTGC-3' (reverse)] and H3F3A[5'-GTACAAAGCAGACTGCCCGCAAAT-3' (forward) and 5'-GTGGATACATACAAGAGAGACTTTGTCCC-3' (reverse)]. Pre-amplification was performed using ABI 2720 Thermal Cycler (Applied Biosystems, Waltham, MA) using 98 °C initial denaturation of 3 minutes, followed by nine cycles of 98 °C for 10 seconds, 60 °C (*IDH1*) or 58 °C (*H3F3A*) for 3 minutes, 72 °C for 30 seconds, and a final 72 °C extension for 2 minutes. The pre-amplified DNA was diluted 1 to 5 with TE buffer.

Digital Polymerase Chain Reaction (ddPCR)

ddPCR was performed using RainDrop Digital PCR system (RainDance Technologies, Billerica, MA). For 50-µL ddPCR reactions, 12 µL of pre-amplified DNA in TE buffer, 1 x TaqMan Genotyping Master Mix (Life Technologies, Carlsbad, CA), 0.2 µM target probes for IDH1[5TET/AGG+TC+GT+CA/ZEN/+TGCT/3IABkFQ (wildtype) and 56FAM/AGG+TC+AT+CA/ZEN/+TGCT/3IABkFQ (mutant)] and H3F3A[5HEX/CA+C+T+C+T+T+GC/3IABkFQ (wildtype) and 56FAM/CA+CT+C+A+T+GCG/3IABkFQ (mutant)], 0.9 µM of forward and reverse primers and 1 x hydrofluorinated droplet stabilizer (RainDance Technologies, Billerica, MA) were added. Tumor tissue DNA and water-only reactions were included as positive-control and negative-control, respectively. Emulsions were prepared on RainDrop Source instrument to produce approximately eight million droplets per 50-µL reaction. Emulsions were then placed on ABI 2720 Thermal Cycler (Applied Biosystems, Waltham, MA) to amplify the targets using initial activation of 95 °C for 10 minutes, followed by 45 cycles of 95 °C for 30 seconds and 60 °C (*IDH1*) or 58 °C (*H3F3A*) for 2 minutes, and in activation of 98 °C for 10 minutes. The reactions were then placed on RainDrop Sense instrument for signal detection.

Supplementary Figures

Figure S1.

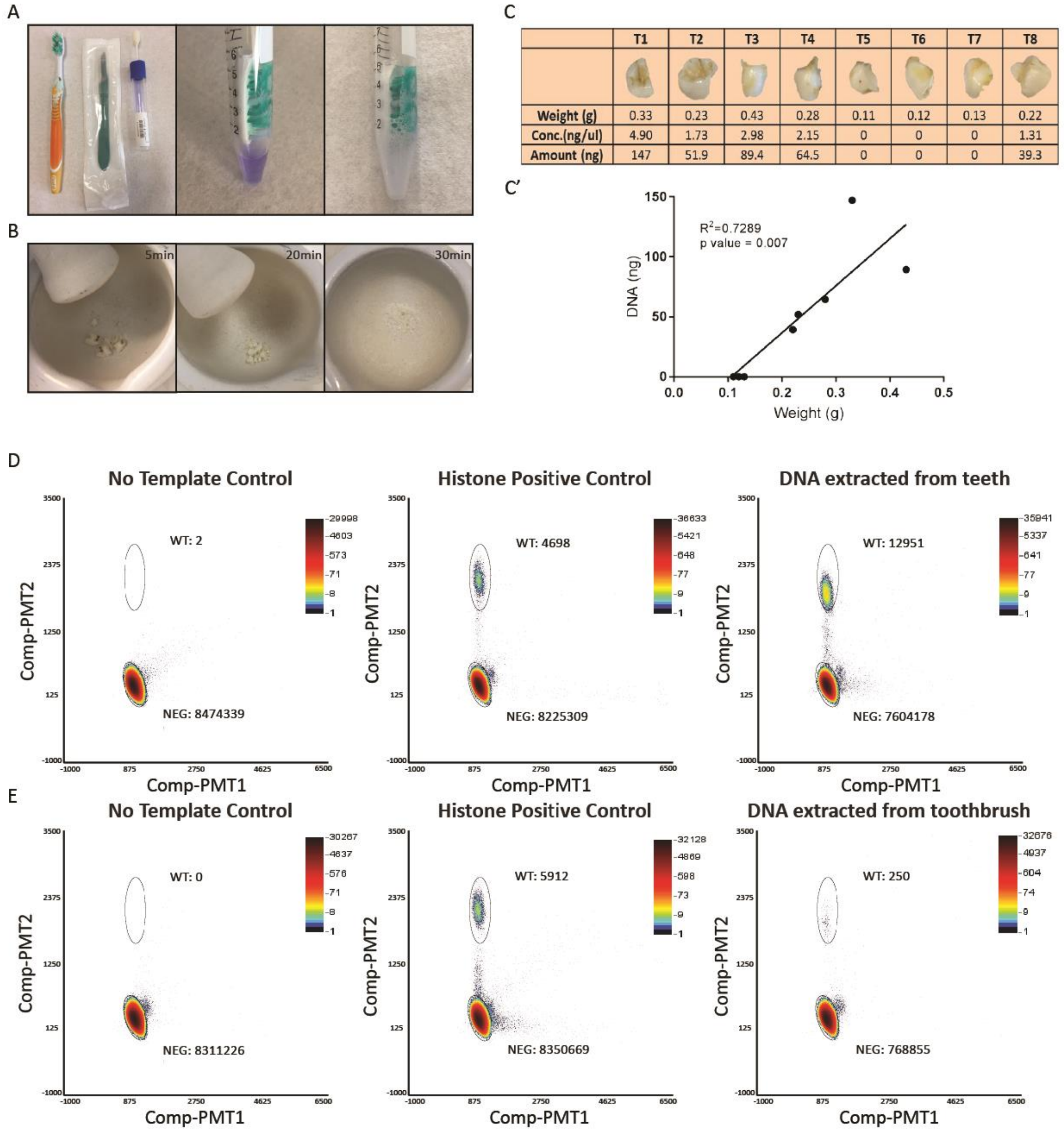

	T1	T2	T3	T4	T5	T6	T7	T8
Weight (g)	0.33	0.23	0.43	0.28	0.11	0.12	0.13	0.22
Conc.(ng/ul)	4.90	1.73	2.98	2.15	0	0	0	1.31
Amount (ng)	147	51.9	89.4	64.5	0	0	0	39.3

Panel A. The toothbrush processing with Tris base solution to isolate DNA from the bristles. **Panel B.** Pulverization of deciduous teeth with liquid nitrogen and mortar and pestle. **Panel C and C'.** Quantification of DNA amount showing linear relationship with tooth weight. **Panel D and E.** ddPCR analysis of DNA isolated from teeth and toothbrush showing presence of wild-type *H3F3A* copies, signifying the feasibility of detecting single nucleotide variation in the DNA.

Figure S2.

A

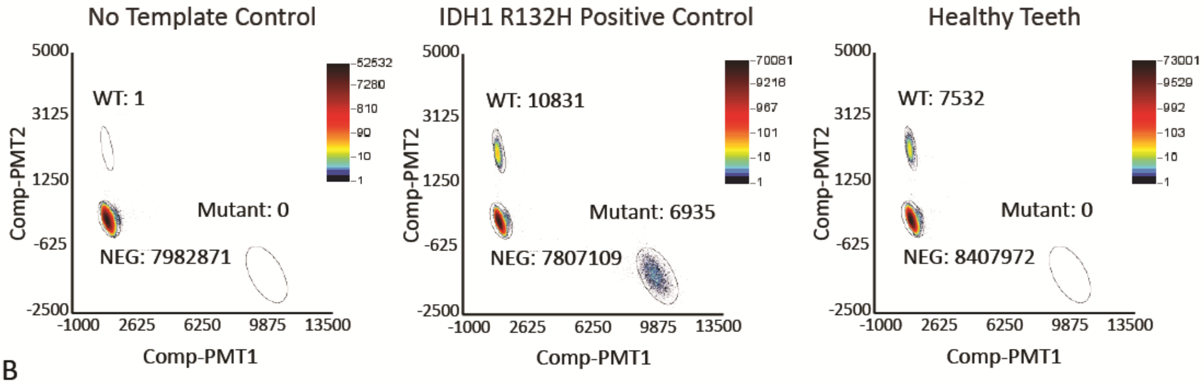

B

Chromosome	Region	Type	Reference	Allele	Zygosity	Count	Coverage	Frequency	Forward read count	Reverse read count	Forward/reverse balance	Average quality	Read count	Read coverage	QUAL
2	208248387..208248388	Deletion	AC	-	Heterozygous	1	5855393	1.70783E-05	1	1	0.5	18.00008337	2	11684400	200
2	208248387..208248388	MNV	AC	CG	Heterozygous	13	5855393	0.000222018	12	2	0.142857143	19.35770413	14	11684400	0.005127796
2	208248387..208248388	MNV	AC	AC	Heterozygous	5837192	5855393	99.68915835	5823721	5824538	0.49996493	37.89831274	11648259	11684400	200
2	208248388	SNV	C	G	Heterozygous	105	5849521	0.001795019	95	80	0.457142857	29.59345296	175	11672697	67.40644907
2	208248388	SNV	C	T	Heterozygous	17177	5849521	0.293647976	17122	17101	0.499693189	35.44553504	34223	11672697	200
2	208248388^208248389	Insertion	-	AAT	Heterozygous	2	5855224	3.41575E-05	1	1	0.5	37.66666667	2	11684213	200
2	208248388^208248389	Insertion	-	T	Heterozygous	2	5855210	3.41576E-05	2	1	0.333333333	18	3	11684185	0.831066024
2	208248388^208248389	Insertion	-	-	Heterozygous	5855220	5855224	99.99993168	5841735	5842473	0.499968419	37.38287171	11684208	11684213	200

C

Chromosome	Region	Type	Reference	Allele	Zygosity	Count	Coverage	Frequency	Forward read count	Reverse read count	Forward/reverse balance	Average quality	Read count	Read coverage	QUAL
2	208248387..208248388	MNV	AC	CT	Heterozygous	4	5188848	7.70884E-05	3	1	0.25	28.25645852	4	10356694	10.42316241
2	208248387..208248388	MNV	AC	GA	Heterozygous	38	5188848	0.00073234	38	1	0.025641026	17.35713104	39	10356694	96.08962008
2	208248387..208248388	MNV	AC	AC	Heterozygous	5187011	5188848	99.96459715	5175907	5177461	0.499924952	37.92045976	10353368	10356694	200
2	208248388	SNV	C	G	Heterozygous	94	5184357	0.001813147	92	82	0.471264368	31.36495886	174	10347727	100.8450204
2	208248388	SNV	C	T	Heterozygous	769	5184357	0.014833083	762	748	0.495364238	31.11380752	1510	10347727	200
2	208248388^208248389	Insertion	-	A	Heterozygous	6	5188759	0.000115635	4	3	0.428571429	33.83333333	7	10356575	20.99505759
2	208248388^208248389	Insertion	-	T	Heterozygous	1	5188759	1.92724E-05	1	0	0	39	1	10356575	1.431756827
2	208248388^208248389	Insertion	-	-	Heterozygous	5188752	5188759	99.99986509	5177625	5178942	0.499936417	37.39758636	10356567	10356575	200

Panel A. ddPCR analysis of DNA isolated from teeth donated by 7 healthy individuals showins absence of IDH1 R132H copies. **Panel B and C.** Next generation Sequencing results using DNA isolated from teeth of sibling A and cheek swab of sibling B, confirming low level mutation allelic frequencies.

Figure S3.

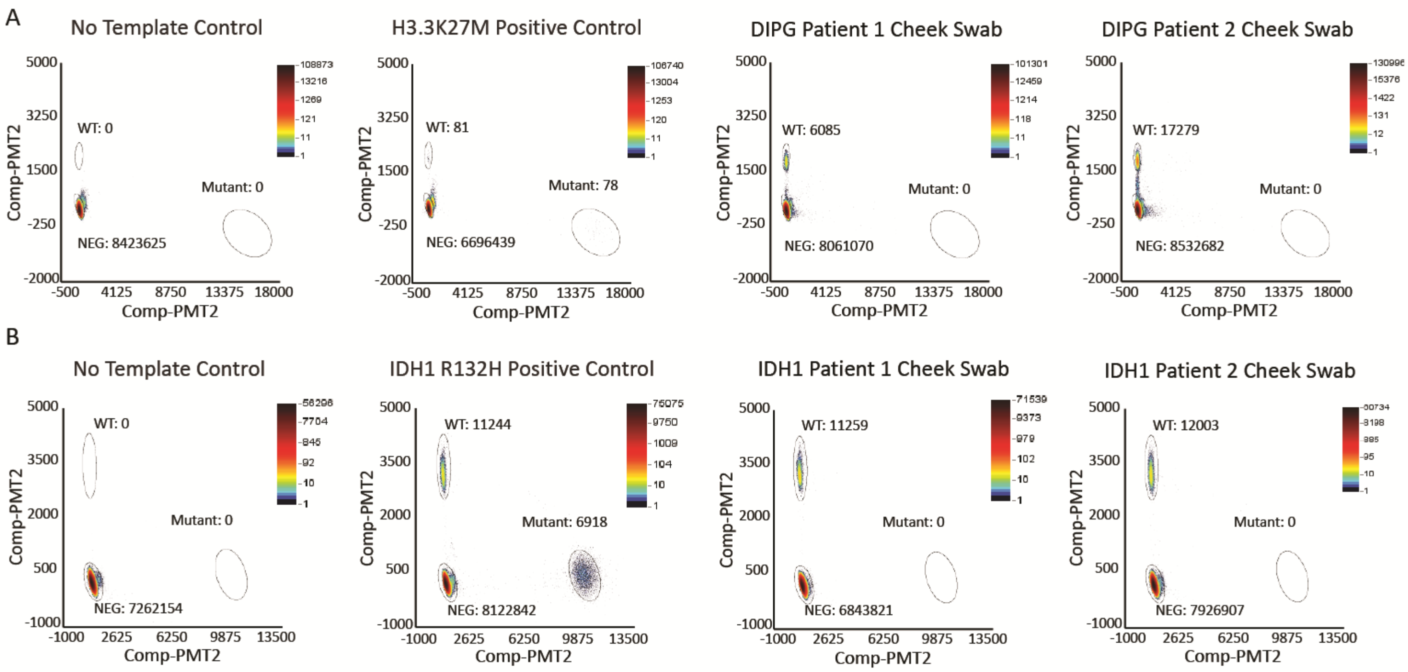

Panel A. ddPCR analysis of DNA isolated from cheek swabs of two DIPG patients showing absence of H3.3 K27M copies, despite positive results in the plasma. **Panel B.** ddPCR analysis of DNA isolated from cheek swabs of two patients diagnosed with IDH1 R132H positive tumors showing absence of IDH1 R132H copies.

Figure S4.

A

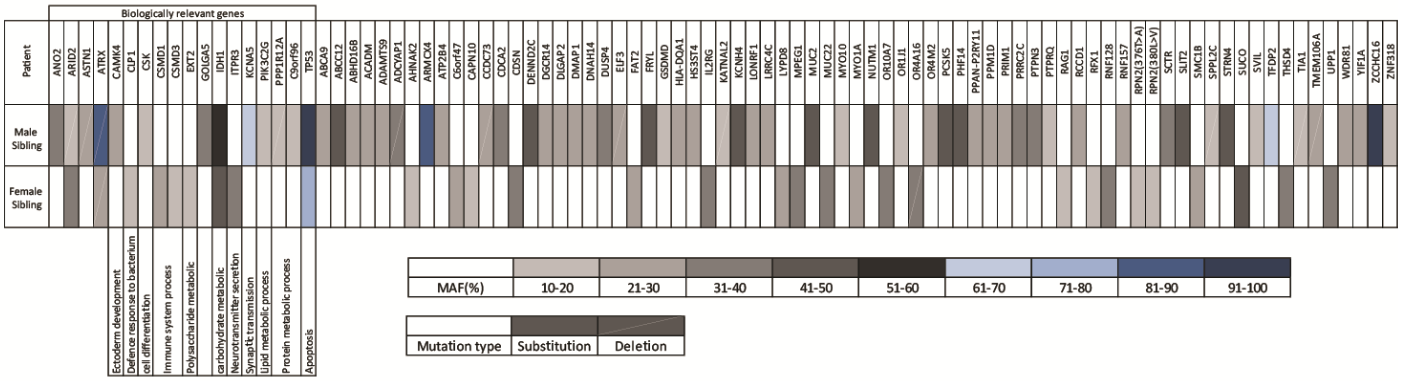

B

Gene	Mutation (AA)	Type	Sequence	Consequence	Mutant reads
ATRX	484R>X	Substitution	TGTTCNTTGTT	Nonsense	90%
IDH1	132R>H	Substitution	AGGTCATCATG	Nonsynoymous coding	58%
TP53	281D>E	Substitution	CGCCGNTCTCT	Nonsynoymous coding	96%
ARID2	G301Afs*27	Deletion	GAGGANGGCAA	Frameshift	17%

C

Gene	Mutation (AA)	Type	Sequence	Consequence	Mutant reads
ATRX	E1450Gfs*15	Deletion	TCCTCNTCTTC	Frameshift	27%
IDH1	132R>H	Substitution	AGGTCATCATG	Nonsynoymous coding	43%
TP53	273R>C	Substitution	AACACNCACCT	Nonsynoymous coding	76%
ARID2	1754R>X	Substitution	TCTTTNGAGAT	Nonsense	38%

Panel A. Results of whole exome sequencing of sibling A and B (using frontal lobe DNA from sibling A and whole blood DNA from sibling B, as healthy control DNA). **Panel B** (sibling A) **Panel C** (sibling B). IDH1 R132H is the only shared mutation in the tumor.
